# Inflorescence temperature influences fruit set, phenology, and sink strength of Cabernet Sauvignon grape berries

**DOI:** 10.3389/fpls.2022.864892

**Published:** 2022-08-15

**Authors:** Markus Keller, Regula Scheele-Baldinger, John C. Ferguson, Julie M. Tarara, Lynn J. Mills

**Affiliations:** ^1^Department of Horticulture, Irrigated Agriculture Research and Extension Center, Washington State University, Prosser, WA, United States; ^2^Department of Agricultural and Food Sciences, Swiss Federal Institute of Technology, Zürich, Switzerland; ^3^United States Department of Agriculture, Agricultural Research Service, Horticultural Crops Research Unit, Prosser, WA, United States

**Keywords:** grape (*Vitis* spp.), temperature manipulation, bloom, fruit set, sink strength, yield components

## Abstract

The temperature during the bloom period leading up to fruit set is a key determinant of reproductive success in plants and of harvest yield in crop plants. However, it is often unclear whether differences in yield components result from temperature effects on the whole plant or specifically on the flower or fruit sinks. We used a forced-convection, free-air cooling and heating system to manipulate the inflorescence temperature of field-grown Cabernet Sauvignon grapevines during the bloom period. Temperature regimes included cooling (ambient −7.5°C), heating (ambient +7.5°C), an ambient control, and a convective control. Cooling significantly retarded the time to fruit set and subsequent berry development, and heating shortened the time to fruit set and accelerated berry development relative to the two controls. Fruit set was decreased in cooled inflorescences, but although the cooling regime resulted in the lowest berry number per cluster, it also decreased seed and berry weight at harvest while not affecting seed number. Cooling inflorescences slightly decreased fruit soluble solids and pH, and increased titratable acidity, but did not affect color density. The inflorescence temperature did not impact leaf gas exchange and shoot growth, and shoot periderm formation occurred independently of the timing of fruit ripening. These results suggest that the temperature experienced by grape flowers during bloom time impacts fruit set and subsequent seed and berry development. Suboptimal temperatures not only reduce the proportion of flowers that set fruit but also limit the sink strength of the berries that do develop after fruit set. Shoot vigor and maturation, and leaf physiology, on the other hand, may be rather insensitive to temperature-induced changes in reproductive development.

## Introduction

Following pollination and fertilization, flowers, or more accurately ovaries, are transformed into fruit in a process known as fruit set. In fruit crops, adequate fruit set is key to attaining high yields for economically profitable production. However, the proportion of flowers that set fruit on a single inflorescence or on a whole plant varies widely. Fruit set in perennial crops such as grapevines can be reduced for many reasons including, for example, abnormal flower development, water or nutrient deficit or excess, pathogen infection, physical injury, insufficient sunlight, or temperatures outside the optimal range (May, 2004; Keller, 2020). Supply of photosynthates, either *de novo* assimilated in the leaves or remobilized from stored reserves in the perennial organs, is often thought to be limiting for fruit set (Vasconcelos et al., 2009; Ruan et al., 2012). In grape flowers, eggs that are not fertilized within 3–4 days after anthesis will degenerate (Kassemeyer and Staudt, 1981). Successful fertilization requires the sequential events of pollination, pollen germination, and pollen tube growth. In grapevines, pollen germination and pollen tube growth are maximal in the temperature range 25°C–30°C, and temperatures <10°C and >35°C inhibit germination, but even temperatures <15°C slow pollen tube growth too much to permit fertilization before eggs degenerate (Staudt, 1982). Excessively high temperatures also decrease pollen tube growth by reducing auxin production in the pistil (Zhu et al., 2021). Therefore, poor fruit set may result from slow pollen tube growth under both cool and hot conditions.

Earlier work on temperature effects on grapevine reproductive behavior was typically conducted under controlled-environment conditions, where whole plants were exposed to different temperature regimes. For example, in a growth chamber study conducted with small, fruiting cuttings, vines exposed to day/night temperatures of 14/9°C from 4 days after budbreak failed to fully differentiate their inflorescences and did not set fruit (Buttrose and Hale, 1973). Though the time to anthesis decreased as the temperature increased, fruit set was highest in vines exposed to 20/15°C and decreased at higher temperatures down to zero at 38/33°C. In a similar experiment, flowering was delayed and fruit set was lower in fruiting cuttings exposed to 12/9°C for 1 week during early bloom compared with vines held at 17/14°C or 25/20°C (Ebadi et al., 1995a). A more recent growth-chamber study with fruiting cuttings concluded that temperature did not influence bloom phenology, but temperatures of 37/31°C (day/night) and higher induced flower abortion (Merrill et al., 2020). Similarly, fruit set decreased in pot-grown grapevines as the growth chamber day temperature during the bloom period increased from 25°C to 40°C, with all night temperatures set at 20°C (Kliewer, 1977). Exposure of pot-grown vines in growth chambers to 40/25°C (day/night) for 4 days during bloom led to complete flower abortion, but the same treatment imposed at fruit set did not alter reproductive growth compared with the 25/15°C control (Greer and Weston, 2010). However, because the growth-chamber approach simultaneously manipulates both sink and source temperatures, it cannot answer the question whether any differences in fruit set or subsequent berry growth arise as a consequence of temperature-induced differences in sink strength of flowers or berries. Sink strength, defined as sink size × sink activity, describes the capacity of a plant organ to use imported resources for growth, metabolism, or storage (Yu et al., 2015).

Previously, we demonstrated that brief periods of shoot apex (i.e., sink) temperature differences during the budswell, budbreak, and early post-budbreak period of grapevines induce persistent, season-long differences in shoot growth and architecture, and reproductive growth (Keller et al., 2010; Keller and Tarara, 2010). At least some of the temperature-induced differences in shoot growth can be attributed to differences in vascular tissue differentiation in the emerging shoots (Galat Giorgi et al., 2020). Here we tested the effects of differences in inflorescence temperature during the bloom period leading up to fruit set. Our main goal was to determine whether differences in inflorescence temperature, rather than whole-plant temperature, were associated with differences in the degree of fruit set and other reproductive traits. We used a forced-convection, free-air cooling and heating system (Tarara et al., 2000) to manipulate the inflorescence temperature of field-grown grapevines without altering the temperature of nearby source leaves or other plant organs. We hypothesized that cooling an inflorescence would reduce its sink strength and heating an inflorescence would enhance its sink strength. Consequently, we expected the cooled inflorescences to show slower development and lower fruit set. But because lower fruit set results in fewer berries per cluster, it was unclear whether a temporary reduction in sink strength at the onset of berry growth would lead to smaller berries by the time of harvest, or whether there would be compensatory berry growth leading to larger berries. Compensatory berry growth was previously observed with the same grape cultivar grown in the same region in which this experiment was conducted, but that study did not test temperature-induced differences in berry numbers (Keller et al., 2008). In addition, sinks and sources are linked through feedback mechanisms, and a decrease in sink activity might result in sugar accumulation and thus lower photosynthesis in source leaves (Körner, 2003; Yu et al., 2015). Assuming that an alteration in the sink strength would lead to a modification in source activity, we also hypothesized that inflorescence cooling would result in a transient decrease in leaf photosynthesis or an increase in shoot growth. We worked with Cabernet Sauvignon (*Vitis vinifera* L.), which is the world’s most widely planted wine grape cultivar, and its reproductive performance is thought not to be especially sensitive to unfavorable environmental conditions (May, 2004). We reasoned that if temperature effects were significant in this cultivar, they would likely also be significant in other, more responsive cultivars.

## Materials and methods

### Vineyard site and treatments

The experiment was conducted in 2004 in a vineyard block of own-rooted Cabernet Sauvignon grapevines planted in 1983 at the Irrigated Agriculture Research and Extension Center near Prosser, WA, United States (46.25°N, 119.73°W, elevation 270 m a.s.l., annual precipitation ~200 mm). The soil is a > 4 m deep Warden fine sandy loam with volumetric water content (*θ_v_*) of ~25% (v/v) at field capacity and ~8% at permanent wilting point (Evans et al., 1993). Vines were planted at 1.8 m within rows and 3 m between rows oriented north–south on a 3% southwest slope. They were trained to bilateral cordons at 1.2 m and winter-pruned to 35–40 buds per vine. A single inflorescence was retained on shoots that were included in the experiment, but no other canopy manipulations were carried out during the growing season. The vineyard was drip-irrigated using regulated deficit irrigation to limit shoot growth after fruit set as described previously (Keller et al., 2005, 2008).

**Figure 1:**
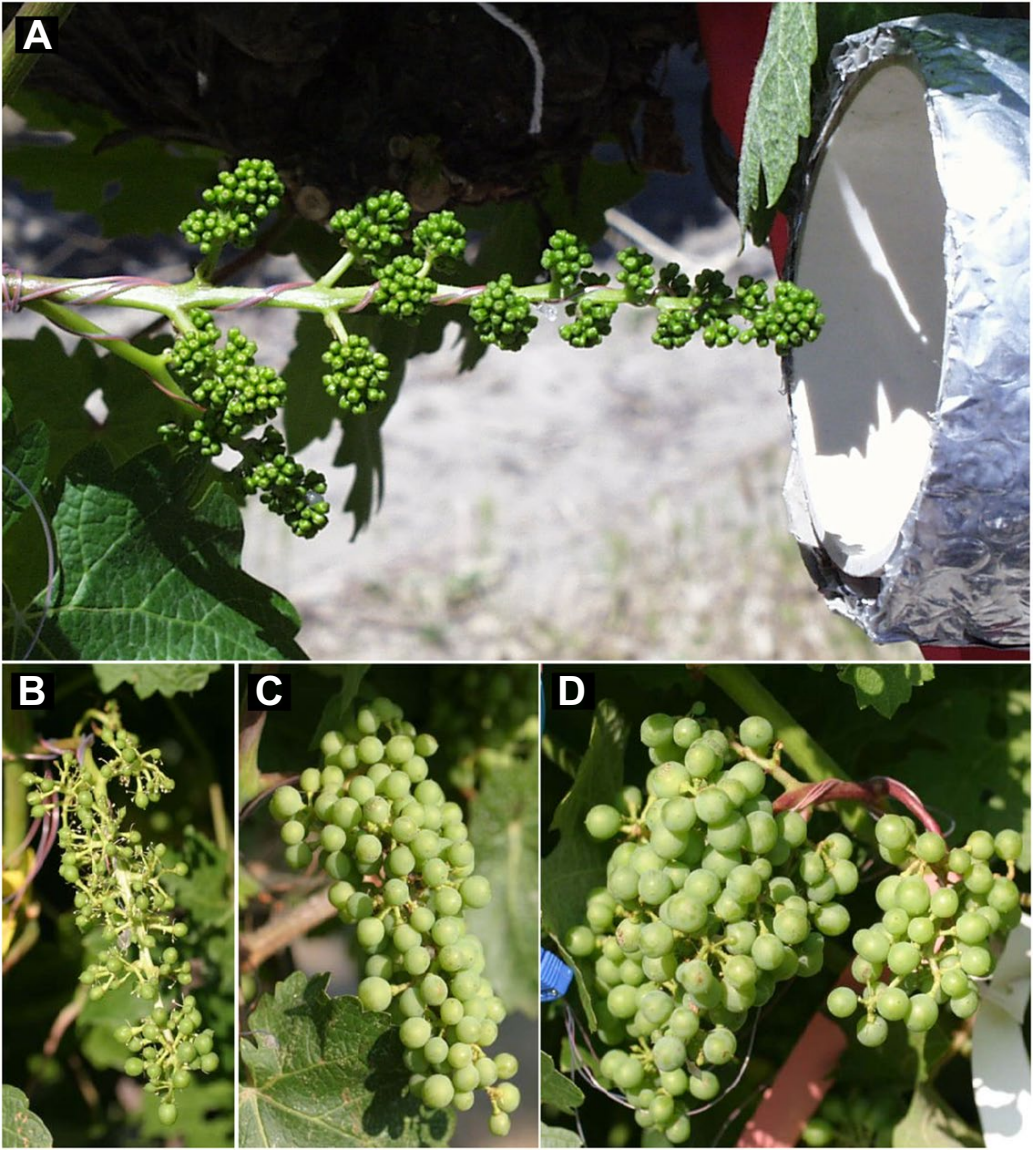
**(A)** Thermocouples embedded in silicon mock flowers and wrapped within an inflorescence, and outlet of cooling tube connected to free-air cooling/heating device used to manipulate inflorescence temperatures during the bloom period of field-grown grapevines. **(B)** Cabernet Sauvignon grape cluster after fruit set following cooling, **(C)** ambient conditions, or **(D)** heating of the inflorescence during the bloom period. Photos in **(B–D)** were taken on the same day immediately after the cooling treatment was terminated.

The temperature of inflorescences was manipulated with a forced-convection, free-air cooling and heating system (Supplementary Figure 1). The system was described by Tarara et al. (2000) and modified as specified in Keller and Tarara (2010). It permits continuous control of target-tissue temperatures without altering solar radiation. Heated or chilled air was delivered directly to individual inflorescences. Four type-T thermocouples (copper-constantan, 0.13-mm diameter, 2-mm junctions), wired in parallel, were embedded in mock flowers made of silicon and wrapped within each inflorescence for temperature measurements (Figure 1A). Control of the cooling/heating system and data acquisition and storage followed the procedures described previously (Keller and Tarara, 2010). Three temperature regimes were applied during the bloom period: ambient, cool (7.5°C below ambient), and warm (7.5°C above ambient). In addition, a convective control (blower without heating or cooling) was included to account for possible effects due to air movement. To prevent heat or chilling injury, the device was programmed to pause at temperatures >40°C or <10°C. Treatments were randomly applied to eight pre-selected inflorescences (*n* = 8), each on a different shoot. Visually uniform (i.e., similar size and architecture) inflorescences were selected after the flowers were fully differentiated prior to the beginning of anthesis. Only inflorescences on the east side of the canopy were selected to minimize sunburn injury on the berries during their subsequent development. Treatments were applied from the beginning of anthesis or cap fall, determined visually as the time of loosening of the first calyptrae, until fruit set, determined visually as the time of initial berry enlargement (Coombe, 1995). While all treatments started on the same day, the duration of temperature control varied as a consequence of phenology-based termination of treatments.

### Environmental data and plant measurements

The *θ_v_* under the vines was measured weekly using a neutron probe (503 DR Hydroprobe; CPN International, Pachero, CA, United States), with six PVC access tubes installed to a depth of 1.2 m both under drip emitters and equidistant between emitters. In addition to the temperature measurements collected by the cooling/heating system, air temperature within and above the canopy, and global irradiance above the canopy, were measured as described elsewhere (Keller et al., 2010; Keller and Tarara, 2010). Growing degree days during the bloom period were calculated from daily minimum and maximum temperatures, applying the standard base temperature for grapevines of 10°C (GDD_10_; Keller, 2020), as well as a base temperature of 15°C (GDD_15_) below which cap fall is thought to cease (May, 2004).

Phenological stages were monitored at least weekly according to the modified E-L system (Coombe, 1995). The length of each treatment shoot was measured weekly until shoot growth ceased, using a tape measure. Numbers of main leaves and of axillary (lateral) shoots and leaves were recorded every time shoot length was measured. Main shoot leaf area was estimated from measurements of midrib length (*L*, in cm) of each leaf, which were converted to leaf area (*A_m_*, in cm^2^) using a regression equation (*A_m_* = 3.44*L* + 0.91*L*^2^, *r* = 0.96, *p* < 0.001) developed from destructive measurements of 200 additional leaves, using a leaf area meter (model LI3100, LiCor, Lincoln, NE, United States). Lateral shoot leaf area (*A*_l_, in cm^2^) was estimated from lateral leaf number (*A*_l_ = 31.23*n* + 2.49*n*^2^, *r* = 0.94, *p* < 0.001). The extent of shoot periderm formation was estimated as the number of brown internodes at the beginning of fruit ripening (termed veraison and defined as 50% of berries having changed color from green to red or blue) and at harvest (Keller and Tarara, 2010).

The length of each treated fruit cluster from its tip to its attachment to the shoot was measured every time shoot length was measured. Flowers were counted using the bagging method, in which inflorescences are enclosed during the bloom period with a fine mesh bag to collect the abscised calyptrae (Kliewer, 1977; Keller et al., 2001; Dry et al., 2010). The abscised calyptrae were weighed and divided by flower counts as a proxy for average flower size (Keller et al., 2010). Some flowers were aborted before they opened and were counted separately. All treated clusters were harvested on the same day once the vineyard exceeded an overall total soluble solids (TSS) target of 24°Brix. The berries of each cluster were counted and weighed, and the flower and berry numbers were used to determine the percentage fruit set. Live green ovaries were excluded from the berry count as suggested by Dry et al. (2010). Additionally, 10 berries per cluster were weighed individually, and their seeds were counted and dried at 65°C for 48 h. The dry seeds were weighed and immersed in water to count “sinkers” and “floaters;” the latter look similar to fully developed seeds (“sinkers”) but are hollow following abortion of their embryo and deterioration of the endosperm several weeks after fruit set (Ebadi et al., 1995a, 1996). Up to 100 berries were collected from each treated shoot to measure fruit TSS, titratable acidity (TA), pH, and color density (i.e., red color due to anthocyanins) as described elsewhere (Spayd et al., 2002). The amount of sugar per berry (sugar content) was estimated from berry weight and TSS.

Leaf gas exchange was measured weekly from the onset of the temperature treatments until 1 week after the cooling treatment was terminated for a total of six measurement dates. Measurements were taken under ambient conditions between 09:00 and 11:00 local standard time, using a CIRAS-2 infrared gas analyzer with a flow rate of 200 ml min^−1^ and a PLC6(U) broad-leaf cuvette (PP Systems, Hertfordshire, United Kingdom) on the leaf above the fruit cluster, which was fully exposed to sunlight (photosynthetic photon flux >1,000 μmol m^−2^ s^−1^) but not to the temperature treatments. Leaf disks were collected 11 days into treatment application from the leaves that were used for gas exchange measurements. Six 0.283-cm^2^ disks were collected from each leaf at sunset, frozen in liquid nitrogen, and stored at −80°C. Non-structural carbohydrates (soluble sugars and starch) were extracted and analyzed as described elsewhere (Halldorson and Keller, 2018).

### Statistical analysis

Data were analyzed using Statistica version 14 (TIBCO Software, Palo Alto, CA, United States). Effects of temperature treatments and changes over time were analyzed by ANOVA; a repeated measures design was used for temporal changes. Duncan’s new multiple range test was used for *post-hoc* means comparisons when treatment effects were significant. The pH values were converted to H^+^ concentrations for data analysis and means were converted back to pH for presentation. Each treated cluster was considered a biological replicate (*n* = 8), and means of the 10-berry samples were used for analysis of the seed data. Results are reported as means ± standard error (SE) unless otherwise specified. Associations between key response variables were tested using Pearson product-moment correlation analysis. Where appropriate, actual *p* values rather than just significance levels are provided.

## Results

### Environment and phenology

**Figure 2:**
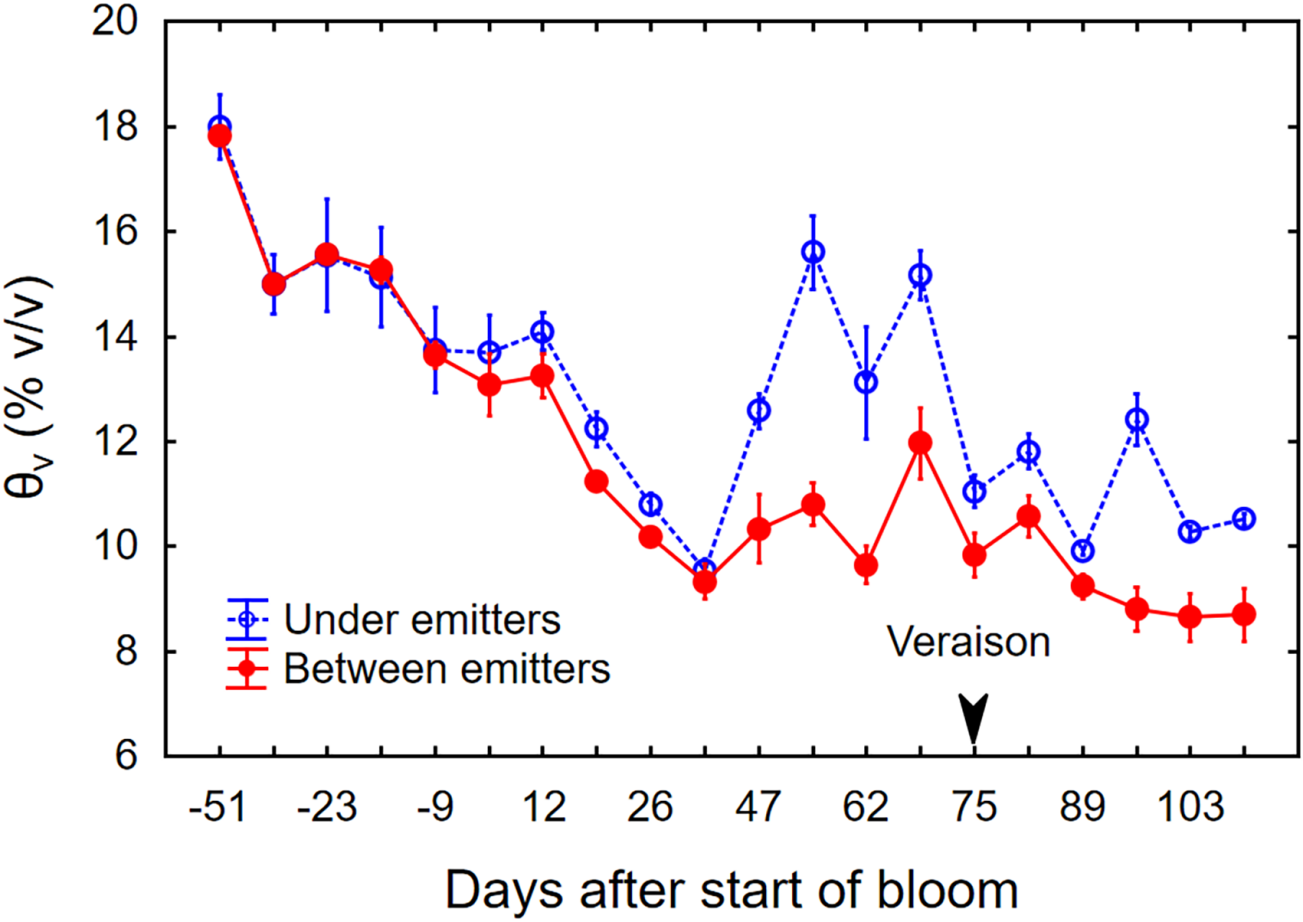
Changes in soil moisture (*θ_v_*) from budbreak through harvest of field-grown, drip-irrigated Cabernet Sauvignon grapevines in southeastern Washington. Data represent the average (±SE) of three separate plants.

At the beginning of the growing season, *θ_v_* under the vines was ~18% (v/v) averaged over the top 90 cm of soil (Figure 2). The *θ_v_* was maintained near 14% during bloom and then allowed to dry down to 9%–10% by mid-July, at which point the berries had reached about pea size and shoot growth slowed. Supplemental drip irrigation then maintained *θ_v_* at 10%–12% between drip emitters and up to 16% beneath emitters through veraison, and near 9% between emitters during fruit ripening. During the experiment, the weather was mostly sunny; global irradiance varied between 0.9 and 1.1 kW m^−2^ at midday, and ambient temperatures ranged from 5°C during the coldest night to 35°C during the warmest day. The grapevine canopy intensified the diurnal temperature fluctuation. Daily minimum temperatures (*T*_min_) inside the canopy were consistently 1°C–2°C lower, and daily maximum temperatures (*T*_max_) were 1°C–2°C higher than ambient temperatures above the canopy (Supplementary Figure 2).

**Figure 3:**
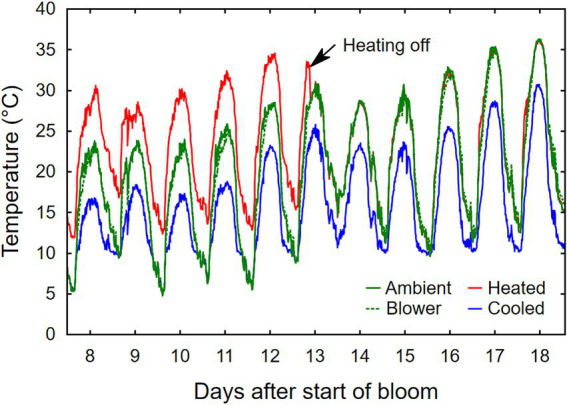
Representative example of temperature profiles of grape inflorescences whose temperature was manipulated, using a free-air cooling/heating device, during the bloom period in the field. Each line represents the average of eight independent inflorescences.

**Figure 4:**
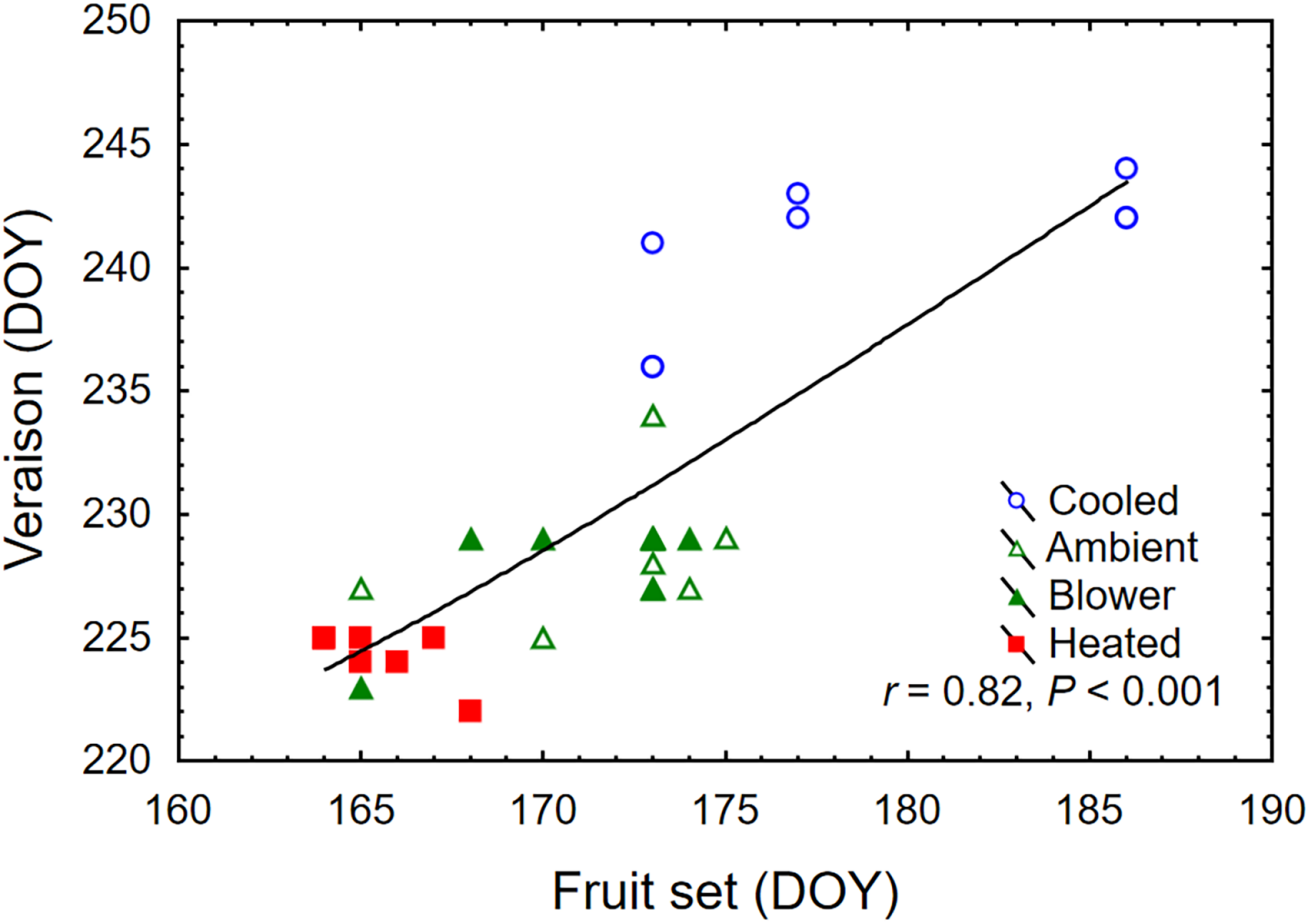
Association between day of year (DOY) of fruit set and veraison of grape clusters whose temperature was manipulated, using a free-air cooling/heating device, during the bloom period in the field. Each treatment was applied to eight independent inflorescences (*n* = 32).

Temperature treatments were applied to inflorescences from the beginning of bloom through fruit set, a period that ranged from 10 days (heated) to 24 days (cooled). Thus heating shortened, and cooling lengthened, the bloom period relative to ambient conditions (Table 1). The treatments generated three distinct diurnal temperature ranges with a difference in average *T*_min_ of 4.9°C and *T*_max_ of 7.9°C between cooled and heated inflorescences (Table 1). During the 13-day heating period, the difference in *T*_max_ was close to the target 15°C, but that in *T*_min_ was lower due to the 10°C treatment cutoff point below which the cooled treatment tracked the ambient control (Figure 3). The temperature of inflorescences in the heating regime peaked at 39°C on 2 days, and the lowest absolute temperature reached in the ambient and cooling regimes was 4.5°C, also on two occasions. Because of generally warmer weather following termination of the heating treatment, the ambient inflorescences subsequently experienced similar temperatures as had the heated inflorescences, and the cooled inflorescences experienced similar temperatures as had the ambient inflorescences (Figure 3). The temperatures of the convective control (blower) were similar to ambient temperatures. During their respective treatment (i.e., bloom) period, heated inflorescences accumulated less GDD_10_ but more GDD_15_ than did cooled inflorescences, while ambient clusters accumulated the most GDD irrespective of the applied base temperature (Table 1). On a daily basis, cooled inflorescences accumulated 6.7 GGD_10_ and 1.7 GDD_15_ and heated inflorescences accumulated 13.1 GGD_10_ and 8.1 GDD_15_ per day (*p* < 0.001), while the two controls were intermediate at 9.5 GDD_10_ and 4.7 GDD_15_. The differences in phenology that arose from the application of the temperature treatments during the bloom period persisted through veraison in mid-August. Compared with ambient and blower control clusters, inflorescence cooling delayed veraison by 13 days and heating advanced veraison by 4 days (Table 1). Moreover, cooling increased, and heating decreased, the variation in the time required to reach subsequent phenological stages. Across treatments, there was a linear relationship between the time of fruit set and the time of veraison (Figure 4).

**Table 1:** Effect of inflorescence temperature regimes generated using a free-air cooling/heating device during the bloom period on bloom duration, average daily maximum (*T*_max_), minimum (*T*_min_), and mean (*T*_mean_) inflorescence temperatures, accumulated growing degree days with base 10°C (GDD_10_) or 15°C (GDD_15_), and time from either first bloom or fruit set to start of ripening (veraison) in field-grown Cabernet Sauvignon grapevines in southeastern Washington.

Treatment	Cooled	Ambient^b^	Blower	Heated	*p*
Bloom duration (d)	24 ± 2.2 a^a^	17 ± 1.1 b	16 ± 1.1 b	10 ± 0.5 c	<0.001
*T*_max_ (°C)	24.2 ± 1.5 c	30.3 ± 1.5 ab	28.7 ± 2.0 b	32.1 ± 2.3 a	<0.001
*T*_min_ (°C)	9.2 ± 0.7 c	10.9 ± 1.3 bc	12.8 ± 1.5 b	14.1 ± 2.0 a	<0.001
T_mean_ (°C)	16.0 ± 1.0 c	21.0 ± 1.2 b	20.2 ± 1.5 b	23.4 ± 1.5 a	<0.001
GDD_10_ (°C)	228 b	263 a	251 a	170 c	<0.001
GDD_15_ (°C)	58 c	133 a	121 a	105 b	<0.001
Bloom to veraison (d)	86 ± 1.1 a	73 ± 0.9 b	73 ± 0.8 b	69 ± 0.4 c	<0.001
Fruit set to veraison (d)	62 ± 1.6 a	56 ± 1.2 b	57 ± 0.8 b	59 ± 0.8 ab	0.009

a Means (±SE) within rows followed by different letters differ significantly (*p* < 0.05, *n* = 8) by Duncan’s new multiple range test.

b Ambient temperatures were averaged for the entire 34-day treatment period (DOY 155–189) because ambient was used to calculate the set point temperatures for cooled and heated treatments.

### Reproductive growth

**Figure 5:**
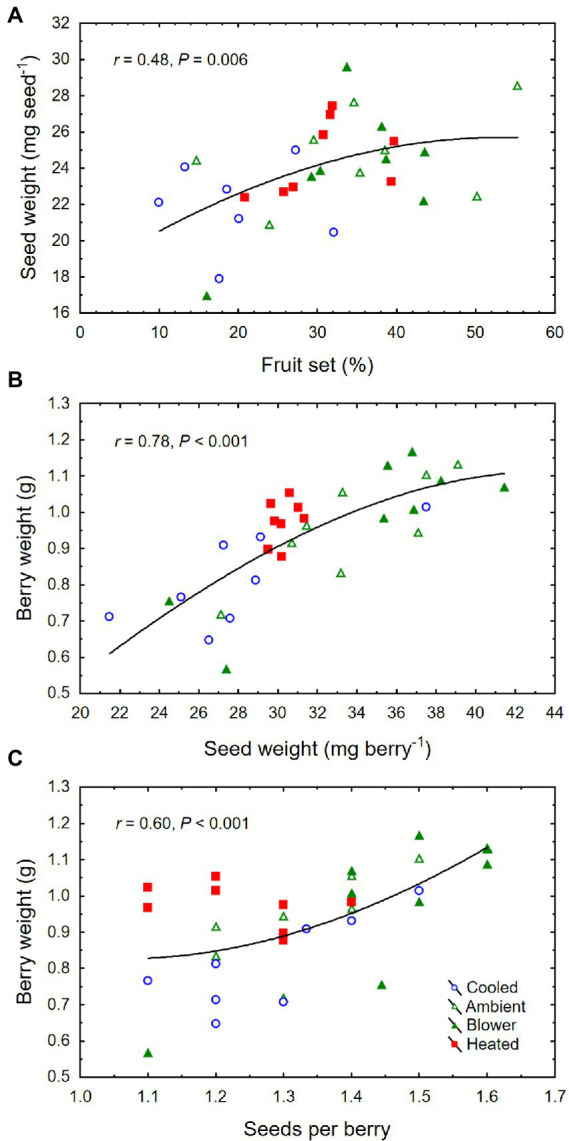
**(A)** Associations between fruit set and mean weight per seed, **(B)** seed weight per berry and berry weight, and **(C)** number of seeds per berry and berry weight at harvest of grape clusters whose temperature was manipulated, using a free-air cooling/heating device, during the bloom period in the field. Each treatment was applied to eight independent inflorescences (*n* = 32).

**Figure 6:**
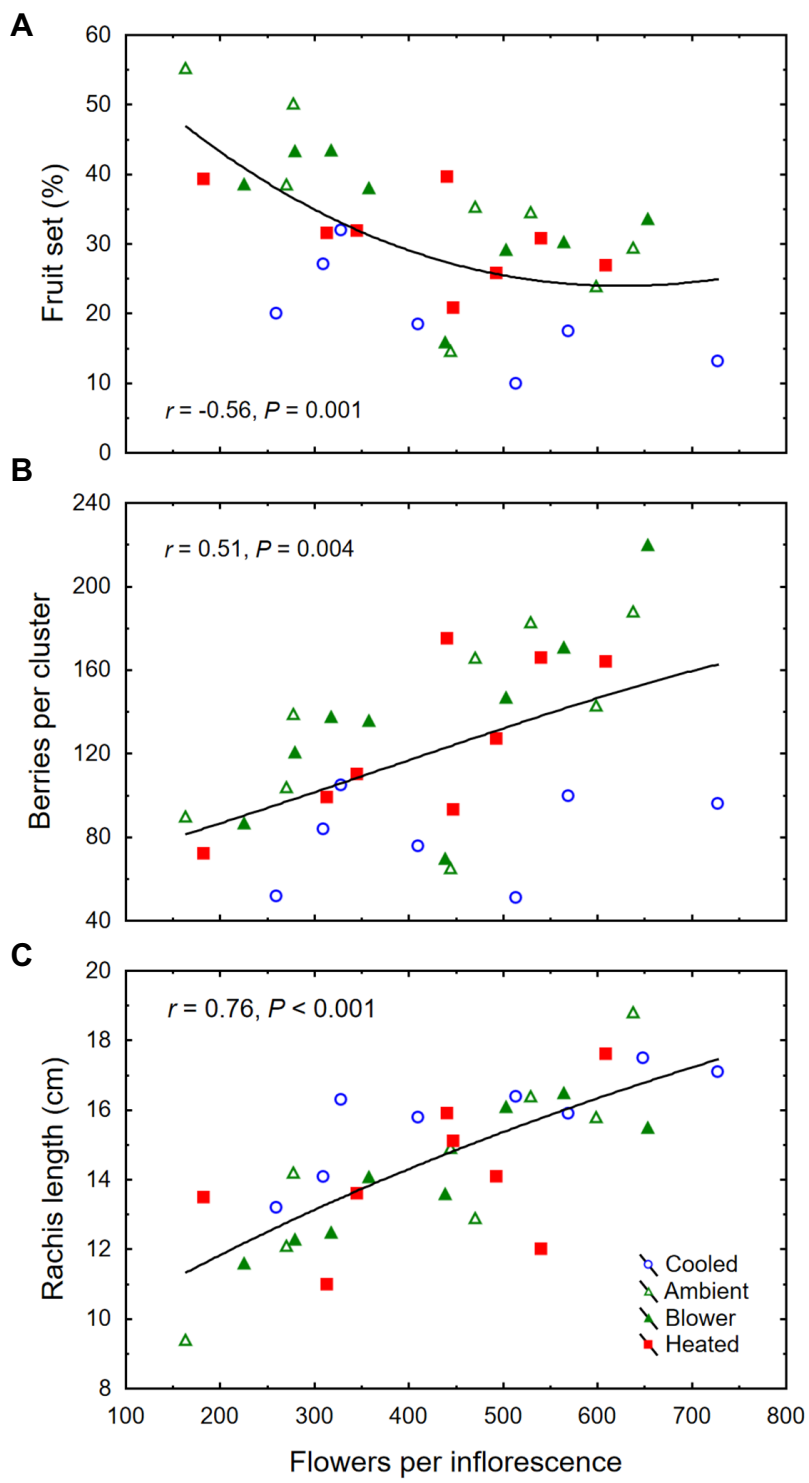
**(A)** Associations between number of flowers per inflorescence and fruit set, **(B)** number of berries per cluster, and **(C)** rachis length at harvest of grape clusters whose temperature was manipulated, using a free-air cooling/heating device, during the bloom period in the field. Each treatment was applied to eight independent inflorescences (*n* = 32).

The inflorescence temperature regimes significantly altered reproductive growth (Figures 1B–D). While the treatments did not affect the average calyptra weight (0.38 ± 0.07 mg; *p* = 0.21), the number of aborted unopened flowers (5.3 ± 1.6; *p* = 0.59) or their percentage (1.4 ± 0.5%; *p* = 0.61), and the number of flowers per inflorescence (433 ± 27; *p* = 0.89), cooling decreased fruit set percentage and the final berry number and weight (Table 2). Heating, however, did not impact these reproductive traits compared with ambient temperatures, which is consistent with the general warming trend during the experiment (Figure 3). On average, 71 ± 3% of the berries had a single seed, 28 ± 3% had two seeds, and 1 ± 1% had three seeds; we found no berries with four seeds and only a single seedless berry. Though there was no clear treatment effect on the number of seeds per berry and no effect on the proportions of sinker and floater seeds (66 vs. 34 ± 3%), inflorescence cooling resulted in the lowest seed weight per berry (Table 2). There also was a positive correlation between fruit set and seed weight (Figure 5A), as well as between seed weight and berry weight (Figure 5B) and between seed number and berry weight (Figure 5C). The latter association illustrates how inflorescence cooling decreased berry weight independently of seed number. Across treatments, fruit set decreased (Figure 6A), and both the number of berries per cluster (Figure 6B) and the rachis length at harvest (Figure 6C) increased as the number of flowers per inflorescence increased. Irrespective of the temperature manipulation treatment applied during bloom, an ~4.5-fold variation in flower number was associated with a 2-fold variation in rachis length. Unlike the flower number, however, the berry number per cluster did not correlate with rachis length (*r* = 0.31, *p* = 0.08), indicating that rachis length was determined before fruit set and that bigger clusters were more compact clusters.

**Table 2:** Effect of inflorescence temperature regimes generated using a free-air cooling/heating device during the bloom period on reproductive growth and harvest fruit composition of field-grown Cabernet Sauvignon grapevines in southeastern Washington.

Treatment	Cooled	Ambient	Blower	Heated	*p*
Flowers per inflorescence	470 ± 60	424 ± 60	417 ± 53	421 ± 48	0.89
Flower size (mg)^a^	0.39 ± 0.01	0.40 ± 0.02	0.37 ± 0.02	0.36 ± 0.01	0.21
Fruit set (%)	20 ± 3 b^b^	35 ± 5 a	34 ± 3 a	31 ± 2 a	0.02
Berries per cluster	81 ± 8 b	135 ± 16 a	136 ± 17 a	126 ± 14 a	0.04
Berry weight (g)	0.79 ± 0.04 b	0.91 ± 0.03 a	0.94 ± 0.06 a	0.96 ± 0.03 a	0.03
Seeds per berry	1.3 ± 0.05 b	1.4 ± 0.05 ab	1.4 ± 0.06 a	1.2 ± 0.04 b	0.03
Floating seeds (%)	34 ± 8	31 ± 5	35 ± 5	34 ± 4	0.96
Total seed weight (mg)	27.9 ± 1.6 b	33.7 ± 1.4 a	34.5 ± 2.0 a	30.3 ± 0.2 ab	0.01
Total soluble solids (°Brix)	23.6 ± 0.2	24.2 ± 0.5	24.3 ± 0.4	24.8 ± 0.3	0.19
Sugar per berry (mg)	185 ± 8 b^b^	221 ± 9 a	229 ± 15 a	237 ± 7 a	0.01
Titratable acidity (g L^−1^)	7.1 ± 0.3 a	5.6 ± 0.3 ab	6.5 ± 0.7 ab	5.3 ± 0.1 b	0.03
pH	3.41 ± 0.02 b	3.58 ± 0.04 a	3.59 ± 0.07 a	3.63 ± 0.02 a	0.01
Color density (AU ml^−1^)	14.7 ± 1.7	14.2 ± 1.4	13.3 ± 1.9	15.1 ± 1.1	0.81

Flowers were counted after bloom, all other data were collected at fruit harvest.

a Average dry weight of the flower calyptrae.

b Means (±SE) within rows followed by different letters differ significantly (*p* < 0.05, *n* = 8) by Duncan’s new multiple range test; absence of letters indicates no significant effect.

**Figure 7:**
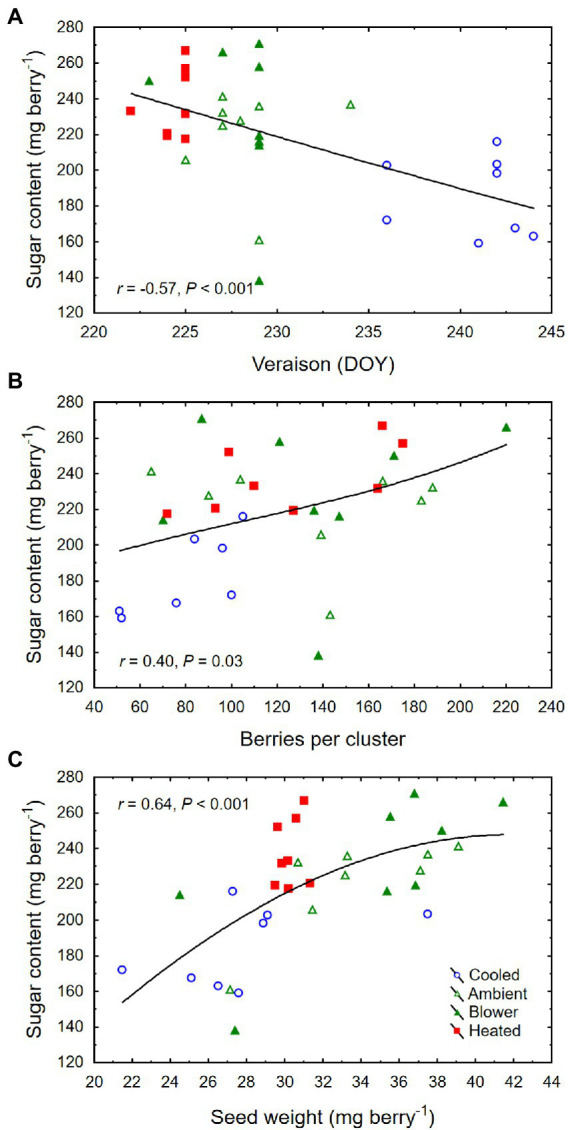
**(A)** Associations between day of year (DOY) of veraison and sugar content per berry, **(B)** number of berries per cluster and sugar content, and **(C)** seed weight per berry and sugar content at harvest of grape clusters whose temperature was manipulated, using a free-air cooling/heating device, during the bloom period in the field. Each treatment was applied to eight independent inflorescences (*n* = 32).

**Figure 8:**
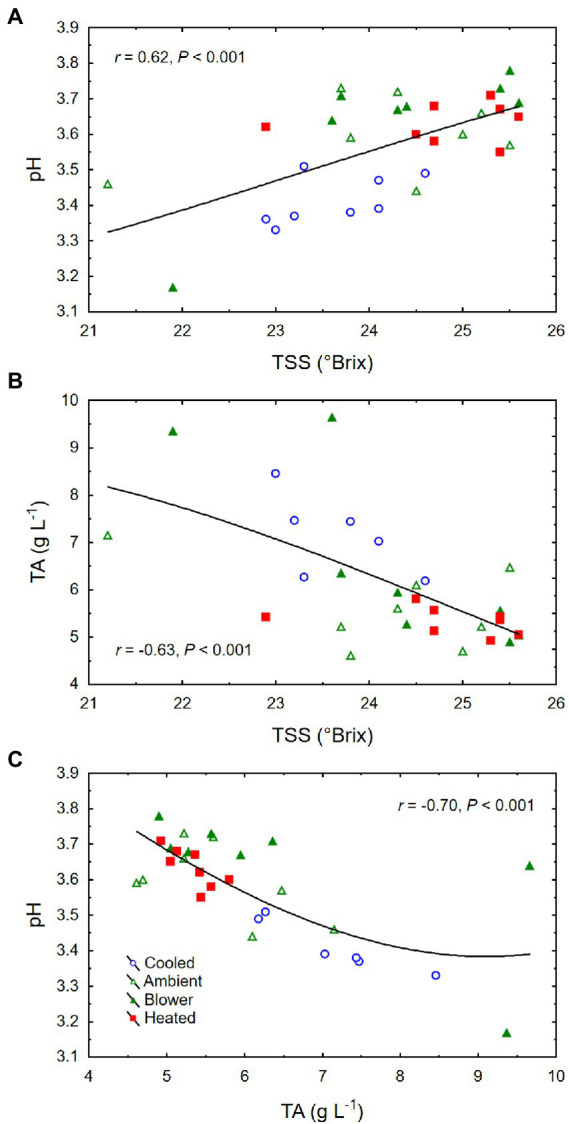
**(A)** Associations between berry total soluble solids (TSS) and pH, **(B)** TSS and titratable acidity (TA), and **(C)** TA and pH at harvest of grape clusters whose temperature was manipulated, using a free-air cooling/heating device, during the bloom period in the field. Each treatment was applied to eight independent inflorescences (*n* = 32).

Though there was no significant treatment effect on berry TSS (i.e., sugar concentration) and color density at harvest, cooling of inflorescences decreased the amount of sugar per berry (i.e., sugar content) and the pH (Table 2). The berries that formed on cooled inflorescences also had higher TA than the berries from heated inflorescences. From veraison to harvest, the berries from all treatments accumulated sugar at similar rates (3.3 ± 0.1 mg d^−1^; *p* = 0.08), but the final sugar content correlated inversely with the date of veraison (Figure 7A). Also, there was a positive correlation between berry number per cluster and final sugar content (Figure 7B), and no correlation between the sugar accumulation rate and the leaf area per unit berry weight (*r* = 0.07, *p* = 0.70). The latter averaged 40 ± 4 cm^2^ g^−1^ across temperature regimes but was higher in the cooling treatment (65 ± 11 cm^2^ g^−1^) than in the other treatments (33 ± 3 cm^2^ g^−1^; *p* = 0.006). Therefore, the lower sugar content of the berries from cooled inflorescences was mostly a result of their shorter ripening period due to delayed veraison, rather than of source limitation. Moreover, as the seed weight per berry increased, so did the sugar content (Figure 7C) but not the sugar concentration (*r* = 0.20, *p* = 0.26). There was a positive correlation between TSS and pH (Figure 8A) and negative correlations between TSS and TA (Figure 8B) and between TA and pH (Figure 8C), all of which were mostly driven by the impact of the temperature regimes applied during bloom (Table 2).

### Vegetative growth

Over the first 4 weeks after bloom, i.e., before shoot growth became very slow due to the declining *θ_v_*, the average shoot growth rate across treatments was 1.6 ± 0.1 cm d^−1^ and the average shoot leaf area expansion rate was 66 ± 3 cm^2^ d^−1^. Many shoot tips died by veraison, and virtually no shoot growth occurred during the berry ripening period. While the leaf area comprised by lateral leaves (i.e., leaves that formed on shoots growing from axillary or prompt buds) made up 23% of the total leaf area at bloom time, this proportion increased to 37% by veraison and remained constant thereafter. Although inflorescence cooling retarded, and heating accelerated, bloom time and berry development compared with ambient conditions, the inflorescence temperature regimes did not impact shoot growth and leaf expansion (Table 3). For instance, both the lateral leaf appearance rate and the shoot leaf area expansion rate were linear functions of the shoot growth rate (*r* = 0.81 and *r* = 0.90, respectively, *p* < 0.001), but all measures of shoot vigor remained unaffected by the temperature regimes. The variation in vegetative growth was greater within than between temperature regimes. Indeed, both the lowest and the highest absolute values of shoot growth rate were found in the heating treatment. Furthermore, none of the reproductive traits correlated with any of the vegetative traits measured here.

**Table 3:** Effect of inflorescence temperature regimes generated using a free-air cooling/heating device during the bloom period on shoot growth and maturation (periderm formation), leaf area expansion, leaf non-structural carbohydrates (NSC), and dormant cane weight of field-grown Cabernet Sauvignon grapevines in southeastern Washington.

Treatment	Cooled	Ambient	Blower	Heated	*p*
Shoot growth rate (cm d^−1^)	1.7 ± 0.1	1.6 ± 0.2	1.4 ± 0.2	1.7 ± 0.3	0.79
Leaf area expansion rate (cm^2^ d^−1^)	64 ± 6	65 ± 8	66 ± 5	69 ± 9	0.97
Leaf NSC (mg g^−1^ fresh weight)					
Starch	36.2 ± 3.1	35.4 ± 3.2	35.8 ± 1.6	31.0 ± 1.7	0.41
Soluble sugars	6.6 ± 0.7	4.8 ± 1.0	5.6 ± 1.0	7.1 ± 1.3	0.41
Shoot length (cm)^a^					
Bloom	84 ± 7	80 ± 4	72 ± 4	87 ± 7	0.32
Veraison	133 ± 10	128 ± 11	115 ± 10	143 ± 15	0.39
Main shoot leaf area (cm^2^)					
Bloom	1,264 ± 126	1,157 ± 68	977 ± 39	1,250 ± 117	0.14
Veraison	2,408 ± 212	2,413 ± 202	2,109 ± 148	2,600 ± 313	0.50
Lateral shoot leaf area (cm^2^)					
Bloom	398 ± 97	353 ± 59	275 ± 42	352 ± 46	0.61
Veraison	1,531 ± 209	1,336 ± 234	1,394 ± 188	1,345 ± 217	0.91
Periderm (brown internodes)					
Veraison	15 ± 0.8 a^b^	8 ± 1.2 b	8 ± 1.0 b	9 ± 0.2 b	<0.001
Harvest	17 ± 1.1	15 ± 1.6	15 ± 1.5	16 ± 1.7	0.79
Dormant cane weight (g)	47.8 ± 9.5	40.7 ± 7.7	37.2 ± 5.4	55.7 ± 13.6	0.54

a All changes over time were tested using a repeated measures design and were significant at *p* < 0.001; the treatment × time interaction was only significant for periderm (*p* = 0.006).

b Means (±SE) within rows followed by different letters differ significantly (*p* < 0.05, *n* = 8) by Duncan’s new multiple range test; absence of letters indicates no significant effect.

At veraison, the shoots with cooled inflorescences had almost twice as many brown internodes as those from the other treatments (Table 3). Because the berries from cooled inflorescences took longer to reach veraison, their shoots had more time to form brown periderm, indicating that periderm formation occurred independently of berry ripening. By harvest, however, the other shoots had caught up and the difference in periderm formation disappeared. Pruning weight measurements in winter revealed no differences in dormant cane weight among treatments (Table 3). The cane weight correlated strongly with the shoot length at veraison (*r* = 0.92, *p* < 0.001).

### Leaf gas exchange and non-structural carbohydrates

**Figure 9:**
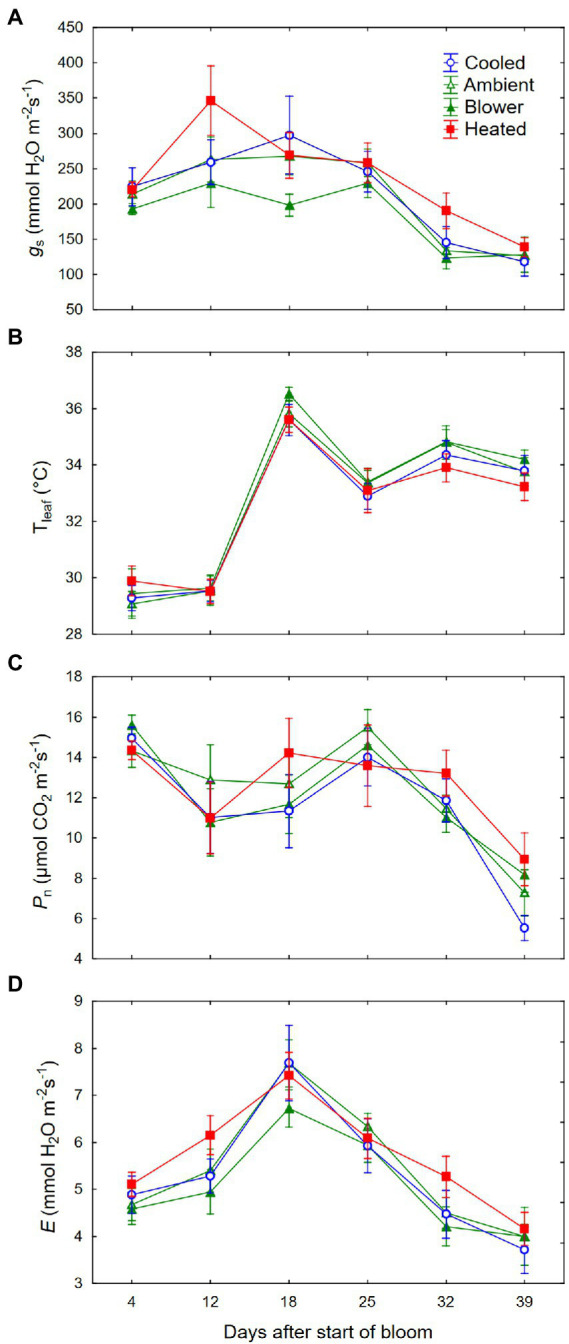
**(A)** Changes in leaf stomatal conductance (*g*_s_), **(B)** leaf temperature (*T*_leaf_), **(C)** photosynthesis (*P*_n_), and **(D)** transpiration (*E*) of grapevine leaves above inflorescences whose temperature was manipulated, using a free-air cooling/heating device, during the bloom period in the field. Each treatment was applied to eight independent inflorescences.

We did not detect a consistent effect of the inflorescence temperature regimes on gas exchange of the leaf above the treated inflorescence. While the temperature treatments were being deployed, leaf stomatal conductance (*g*_s_) tended to be higher in the heating regime than in the blower control (Figure 9A). This difference was not caused by changes in leaf temperatures (T_leaf_) induced by the inflorescence treatments; *T*_leaf_ was similar across all temperature regimes (*p* = 0.39; Figure 9B). Moreover, neither the heating treatment nor the blower control differed from the ambient control and the cooling regime, and although the overall temperature effect on *g*_s_ was significant (*p* = 0.008), this did not apply to any individual day. Despite the differences in *g*_s_, net photosynthesis (*P*_n_) did not respond to the temperature regimes (*p* = 0.49; Figure 9C). The treatments also did not affect transpiration rates (*p* = 0.11; Figure 9D). No significant differences were found after the treatments were terminated, and *g*_s_ and *P*_n_ decreased in response to the declining *θ_v_*. Whereas *E* was closely correlated with *g*_s_ (*r* = 0.83, *p* < 0.001), the correlation between *P*_n_ and *g*_s_, though significant, was quite low (*r* = 0.23, *p* = 0.002). Leaf non-structural carbohydrates at sunset were dominated by starch (79.7 ± 1.4%), followed by glucose (15.1 ± 0.5%), fructose (3.0 ± 0.2%), and sucrose (2.3 ± 0.4%). Neither starch nor soluble sugars differed among inflorescence temperature regimes (Table 3). There was no correlation between starch and sugar concentrations (*r* = −0.03, *p* = 0.86). There also was no correlation between *P*_n_ and either leaf starch (*r* = −0.17, *p* = 0.37) or soluble sugars (*r* = 0.08, *p* = 0.67).

## Discussion

The present study, conducted in a vineyard in arid southeastern Washington, United States, found that the temperature of Cabernet Sauvignon inflorescences during the bloom period may alter flowering phenology, fruit set, and subsequent cluster development independently of the temperature of the canopy. Using targeted manipulation of inflorescence temperatures in canopies exposed to ambient temperatures, we found that warm microclimatic conditions were favorable for fruit set and seed development, which in turn advanced berry development and increased final berry weight. Flowers and berries on cooled inflorescences reached the subsequent phenological stages significantly later than those on heated inflorescences and produced smaller berries. Thus, manipulating the inflorescence temperature not only altered the duration of the bloom period (i.e., time to fruit set) but also had a lasting effect on berry development after fruit set. The time from fruit set to veraison varied relatively little among treatments, suggesting that differences in later phenology arose mostly from differences in the duration of bloom. By contrast, the temperature treatments had no effect on the number and size of the flowers, since the treatments were applied only after the flowers had been fully differentiated (Keller, 2020).

Cooling inflorescences to an average *T*_max_/*T*_min_ of ~24/9°C not only lengthened the bloom period by 7 days but also reduced fruit set. However, despite the lower number of berries per cluster resulting from reduced fruit set, those clusters also had smaller berries with lighter seeds and lower sugar content at harvest, indicating that there was no compensatory increase in berry growth or sugar accumulation. In a growth chamber study, fruiting cuttings exposed to 14/9°C (day/night) failed to fully differentiate their inflorescences and did not set fruit (Buttrose and Hale, 1973). Fruit set in that study was highest in plantlets exposed to 20/15°C and decreased at higher temperatures down to zero at 38/33°C. Elsewhere, fruit set, seed number, and subsequent cell division and berry growth were highest when vines were held during bloom at 25/20°C, and decreased at higher temperatures (Kliewer, 1977). Another growth chamber study found no effect on fruit set but a reduction in seed number per berry in vines grown during bloom at 15/10°C compared with 25/10°C or 25/20°C (Ewart and Kliewer, 1977). In our study, inflorescence heating to an average *T*_max_/*T*_min_ of ~32/14°C shortened the bloom period by 7 days but did not reduce fruit set. Except in the cooled inflorescences, fruit set in our study was similar to that observed previously for Cabernet Sauvignon in the same vineyard (Keller et al., 2010) and in different Australian wine regions but lower than in eight of the 10 cultivars tested in the Australian study (Dry et al., 2010). Differences in reproductive performance and environmental plasticity among grape cultivars is one possibility for differences among experiments. However, contrary to the cooling treatment, our heating treatment also decreased the variability in subsequent seed and berry development, suggesting that the high-temperature regime used here was close to the optimum for reproductive growth of Cabernet Sauvignon.

The fact that our experiment was conducted with a single grape cultivar in a single season limits the conclusions that can be drawn from our results. For example, this study could not identify an upper-temperature limit during bloom time, likely because the ambient *T*_max_ of inflorescences exceeded 35°C on only three consecutive days (peaking at 36.3°C) starting 4 days after the heating regime was terminated. A recent review of high-temperature effects on plant performance concluded that shortening the time to fruit set due to moderately high temperatures reduces seed and fruit weight (Zhu et al., 2021). Evidently, the temperature regime imposed by our heating treatment was not high enough to impair these reproductive traits. Despite shortening the bloom period, the moderately high temperatures applied in this study did not conclusively reduce seed weight and had no effect on final berry weight. The temperature response of Cabernet Sauvignon reproductive growth observed here resembles that of the subtropical crop tomato (*Solanum lycopersicum* L.) rather than that of the temperate (cereal) crops reviewed in Zhu et al. (2021). Even higher temperatures, however, may be detrimental to grapevine reproductive performance. For example, temperatures above 33/27°C (day/night) led to flower abortion and poor fruit set in growth chamber experiments (Buttrose and Hale, 1973; Greer and Weston, 2010; Merrill et al., 2020). Increasing the canopy temperature by 2°C–4°C above ambient during bloom in the field also reduced fruit set, and fruit set was lower in the warmer (*T*_max_ ≈ 33°C) of the 2 years studied (Pagay and Collins, 2017). The diurnal temperature range reached under field conditions in our study (15°C–20°C) was greater than that imposed in earlier growth-chamber experiments (3°C or 5°C in Ebadi et al., 1995a,b, 5°C or 12.5°C in Buttrose and Hale, 1973; 6°C in Merrill et al., 2020; 10°C or 15°C in Greer and Weston, 2010) but similar to other field experiments (Pagay and Collins, 2017).

Our forced-convection, free-air cooling and heating system worked well in that the desired temperature patterns were achieved, set points were not exceeded, and differences between the ambient (i.e., non-treated) control and the convective (i.e., blower) control were insignificant. The system thus served as an effective tool to manipulate the inflorescence microclimate, just as it had previously been used to manipulate the cluster microclimate during fruit development and ripening (Spayd et al., 2002). Moreover, the temperature regimes applied to inflorescences did not alter shoot growth, leaf gas exchange, and leaf non-structural carbohydrates. Gas exchange and carbohydrate measurements were conducted on the leaf above each treated inflorescence, because during bloom time this leaf is a main source of photosynthates for the inflorescence below it, and is thus most likely to be influenced by source–sink feedback networks (Keller, 2020). Consequently, the manipulation of sink strength by our temperature regimes did not modify the strength of nearby sources or the strength of other sinks (e.g., growing shoot tips). In addition, the reduction in fruit set, as well as seed and berry size following inflorescence cooling was not a result of limited photosynthate supply from the leaves but instead was a consequence of temperature acting on the flowers themselves. In growth chambers, low temperatures (12/9°C) just before and during bloom were found to impair embryo sac development in grape ovaries, possibly leading to an increase in floater seeds with aborted embryo and endosperm (Ebadi et al., 1995a,b). In our field experiment, however, there was no evidence that the number of seeds per berry or their viability was influenced by inflorescence temperature; the proportion of floater seeds was the same across all treatments. The proportion of floaters was similar to that reported for Chardonnay seeds by Ebadi et al. (1995a, 1996) but much higher than in a previous study with Cabernet Sauvignon, yet the seed number and seed weight were similar to that study (Hall et al., 2011). Both pollen germination on the stigma and the rate of pollen tube growth are also temperature-dependent; at <15°C these processes often become too slow for fertilization to occur (Staudt, 1982; Ebadi et al., 1995b). In some cases, pollen was sterile when inflorescences experienced temperatures <15°C at the beginning of bloom, even when photosynthate supply was abundant (Koblet, 1966). On the other hand, pollen tube growth is readily reactivated when warm temperatures follow a brief (e.g., 2 days) episode of cool temperatures (Staudt, 1982). In our experiment, the temperature of cooled inflorescences fluctuated on a diurnal and longer-term basis but was not consistently below 15°C, and thus, fruit set was reduced but not prevented entirely. In addition to the processes related to fruit set, inflorescence cooling likely also reduced cell division and cell expansion in the resulting berries, thus decreasing their final size. Both of these processes occur simultaneously during the first few weeks after anthesis (Harris et al., 1968), and both are limited by cold stress (<15°C) and heat stress (>35°C; Hale and Buttrose, 1974; Kliewer, 1977). We did not evaluate the number and size of mesocarp cells here, but because flowering occurs asynchronously across an inflorescence, the extended bloom period in the cooling treatment implies that berries resulting from early fruit set within a cooled inflorescence continued to be exposed to low temperatures after fruit set until the last flowers set fruit and cooling was terminated.

While the impact of inflorescence cooling on berry growth (i.e., cell division and cell expansion) decreased berry weight independently of berry number per cluster and seed number per berry, the final berry weight also correlated with both seed number and total seed weight across treatments. The correlation between seed number or weight and berry size is well established and is related to the seeds releasing growth-promoting auxin and gibberellin after fruit set (Winkler and Williams, 1935; Olmo, 1946; Keller, 2020). While only a single seed is required for a grape berry to develop, the total seed weight correlates strongly with the growth rate and final size (i.e., sink strength) of mesocarp cells and hence berries (Friend et al., 2009). Additionally, the correlation between final rachis length and flower but not berry number observed here supports the idea that developing flowers, much more so than berries, determine rachis growth and development (Theiler and Coombe, 1985; Gourieroux et al., 2016). Whether the connection between flower number and rachis growth is causally related to the occurrence of a ripening disorder named bunch-stem necrosis (BSN) remains unknown. An inverse correlation between the average bloom-time *T*_max_ and the incidence of late-season BSN has been reported for several grape cultivars (Theiler and Müller, 1987). However, although Cabernet Sauvignon is generally susceptible to BSN (Hall et al., 2011), we found no symptomatic clusters in the present study.

Differences in phenology, berry numbers, and berry size following inflorescence temperature manipulation did not alter shoot growth. Apparently, these shoots were not sink-limited. In earlier work with field-grown grapevines, reproductive growth was positively correlated with vegetative growth within shoots (Keller et al., 2015). Nonetheless, many wine industry members believe that late harvest or delayed fruit ripening will slow down shoot maturation and compromise the cold hardiness of overwintering buds. Our results do not support this assumption. Counting the number of brown internodes per shoot showed that the degree of periderm formation (often incorrectly termed “lignification”) was independent of fruit development. The timing of veraison (i.e., of ripening initiation) did not coincide with the formation of periderm across treatments. The former was strongly altered by the temperature regimes applied during the bloom period, whereas the latter occurred simultaneously across treatments, which shows that ripening initiation and periderm formation are independent processes. This result is similar to the finding that veraison does not coincide with the transition of grapevine buds from paradormancy to endodormancy (Camargo-Alvarez et al., 2020). In both studies, the timing of veraison was highly responsive to temperature, whereas periderm formation and bud dormancy seemed to be driven mostly by photoperiod.

Grape growers have several options for influencing the microclimate of an inflorescence or cluster. Examples include the choice of training system, trunk height, vineyard floor management (e.g., control of weeds or cover crops), and canopy management (e.g., manipulation of the number of leaves around the clusters). Different training systems result in differences in canopy density and shoot orientation, which influences the microclimate around the clusters (Reynolds and Vanden Heuvel, 2009). Under the climatic conditions of the present study, a vertical temperature gradient of up to +4°C m^−1^ may exist upwards from the cooler trunk base (Peña Quiñones et al., 2019). An inflorescence that grows close to the ground is therefore likely to be exposed to cool air more often than is an inflorescence at a greater distance from the ground. Additionally, bare soil can be a source of heat transfer to the inflorescences by convection, especially during sunny days and when the soil surface is dry. Thus inflorescences are likely to be cooler in vineyards with a floor cover of weeds or cover crops. Removing leaves to expose clusters to solar radiation will also result in higher daytime (but somewhat lower nighttime) temperatures of inflorescences. However, leaf removal at or before the time of bloom eliminates source area and generally reduces fruit set (Vander Weide et al., 2021). Shoot thinning (i.e., removal of entire shoots) early in the growing season also reduces the density of the canopy and thus probably contributes to a warmer microclimate around the clusters (Wang et al., 2019).

## Conclusion

The forced-convection, free-air cooling and heating system is a useful research tool to investigate sink/source interactions and to study effects of organ temperature independently from those of light. By manipulating grape inflorescence temperatures, rather than whole-plant temperatures, we found that sink strength and hence fruit development is dependent on the temperature acting directly on the sinks themselves. Our results also showed that temperature differences perceived by flowering structures before fruit set can alter the subsequent phenological development of the fruit. Cooling inflorescences from the beginning of bloom lengthened, and heating inflorescences shortened, the bloom period, thus delaying (cooling) or accelerating (heating) the time to fruit set as well as the time to ripening onset. Among grapevine yield components, both berry number per cluster and final berry weight were reduced when the inflorescences experienced cool temperatures. At harvest, berries developing from cooled inflorescences also had lower total seed weight and sugar content, and higher acidity. Whereas daily average temperatures above 16°C appear to be necessary during the bloom period to maximize fruit set and subsequent berry development in Cabernet Sauvignon grapevines, no upper-temperature limit was found in this study. Though our highest temperature regime (daily average 23.4°C) did not modify fruit set and berry traits compared with the ambient regime (daily average 21°C), it did reduce the time to fruit set and veraison, and decreased the variability in subsequent berry development and seed weight, suggesting that this regime was close to the optimum for reproductive performance. The applicability of these results to other grape cultivars, especially cultivars that differ in their sensitivity to temperature variation, remains to be tested.

## Data availability statement

The raw data supporting the conclusions of this article will be made available by the authors, without undue reservation.

## Author contributions

MK conceived of the study, obtained the funding, and wrote the manuscript. JF and JT designed and operated the free-air cooling and heating system. RS-B, JF, and LM conducted the experiment. RS-B and MK analyzed the data. All authors contributed to the article and approved the submitted version.

## Funding

This research was funded by the USDA Northwest Center for Small Fruits Research (grant number 5953582718), Washington State Grape and Wine Research Program, and Walter Hochstrasser Stiftung.

## Conflict of interest

The authors declare that the research was conducted in the absence of any commercial or financial relationships that could be construed as a potential conflict of interest.

## Publisher’s note

All claims expressed in this article are solely those of the authors and do not necessarily represent those of their affiliated organizations, or those of the publisher, the editors and the reviewers. Any product that may be evaluated in this article, or claim that may be made by its manufacturer, is not guaranteed or endorsed by the publisher.
