# Supplementary figures and images for: Inflorescence temperature influences fruit set, phenology, and sink strength of Cabernet Sauvignon grape berries
Source: Front Plant Sci. 2022 Aug 15;13:864892. doi: 10.3389/fpls.2022.864892 (PMC9420974; doi:10.3389/fpls.2022.864892)

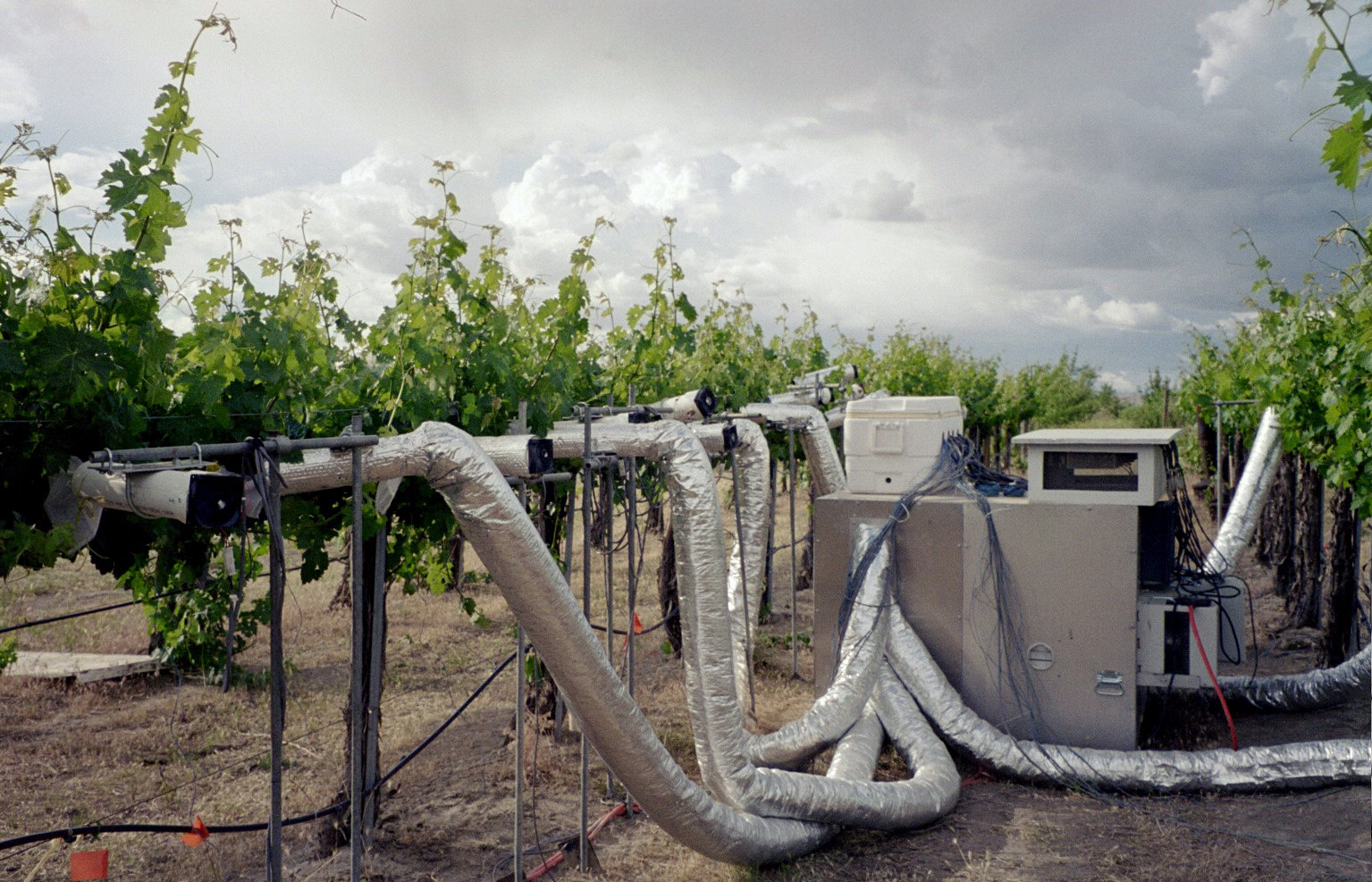

Supplement: Supplementary file 1 [file Image_1.TIF]

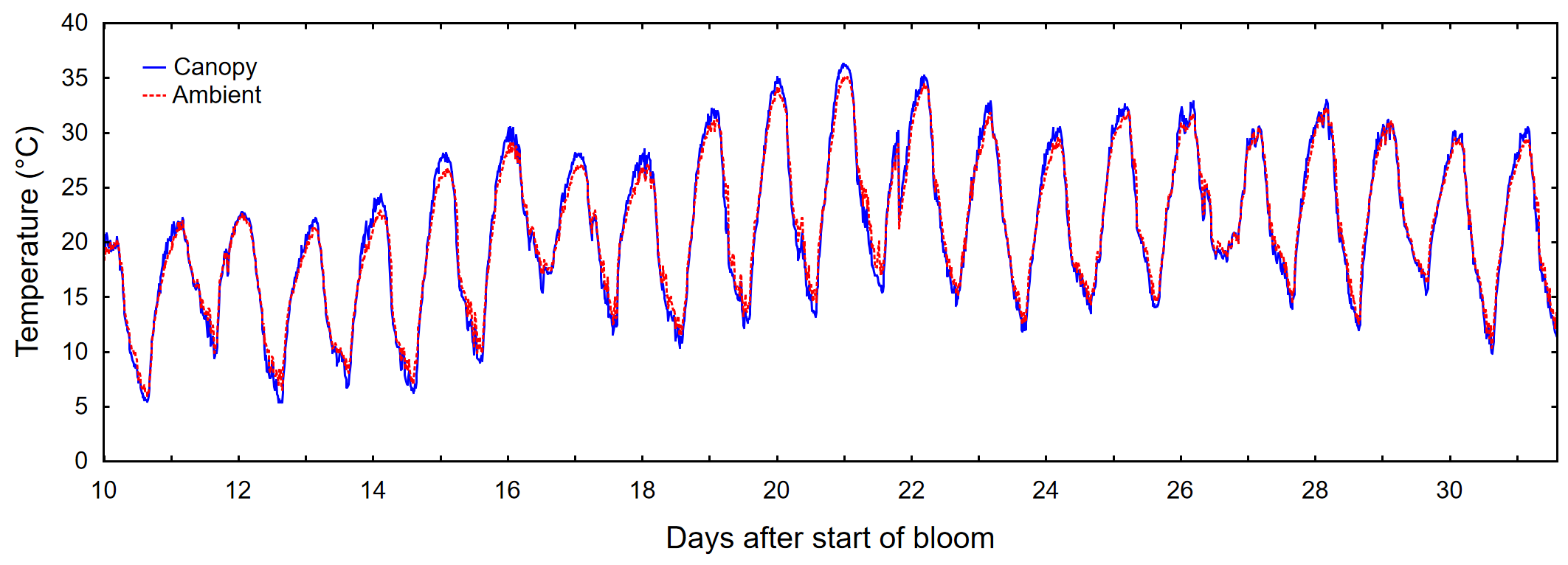

Supplement: Supplementary file 2 [file Image_2.TIF]
